# Rapid, simple, and clinically applicable high-performance liquid chromatography method for clinical determination of plasma colistin concentrations

**DOI:** 10.1186/s40780-018-0119-x

**Published:** 2018-08-20

**Authors:** Yuki Hanai, Kazuhiro Matsuo, Takayoshi Kosugi, Ayumu Kusano, Hayato Ohashi, Itsuki Kimura, Shinobu Hirayama, Yuta Nanjo, Yoshikazu Ishii, Takahiro Sato, Taito Miyazaki, Kenji Nishizawa, Takashi Yoshio

**Affiliations:** 1 Department of Pharmacy, Toho University Omori Medical Center, 6-11-1 Omori-nishi, Ota-ku, Tokyo, 143-8541 Japan; 2 Department of Clinical Pharmacy, Faculty of Pharmaceutical Sciences, Toho University, 2-2-1, Miyama, Funabashi, Chiba, 274-8510 Japan; 3 Department of Pharmacy, National Defense Medical College Hospital, 3-2 Namiki, Tokorozawa, Saitama, 359-8513 Japan; 4 Department of Microbiology and Infectious Diseases, Toho University School of Medicine, 6-11-1, Omori-nishi, Ota-ku, Tokyo, 143-8541 Japan; 5 Department of General Medicine and Emergency Care, Toho University School of Medicine, 6-11-1, Omori-nishi, Ota-ku, Tokyo, 143-8541 Japan

**Keywords:** Colistin, High-performance liquid chromatography, Fluorescence detection, 9-fluorenylmethyl chloroformate, Therapeutic drug monitoring, Haemodialysis

## Abstract

**Background:**

Since both the antibacterial effects and common adverse effects of colistin are concentration-dependent, determination of the most appropriate dosage regimen and administration method for colistin therapy is essential to ensure its efficacy and safety. We aimed to establish a rapid and simple high-performance liquid chromatography (HPLC)-based system for the clinical determination of colistin serum concentrations.

**Methods:**

Extraction using a solid-phase C18 cartridge, derivatisation with 9-fluorenylmethyl chloroformate, and elution with a short reversed-phase Cl8 column effectively separated colistin from an internal standard. The HPLC apparatus and conditions were as follows: analytical column, Hydrosphere C18; sample injection volume, 50 μL; column temperature, 40 °C; detector, Shimadzu RF-5300 fluorescence spectrophotometer (excitation wavelength, 260 nm; emission wavelength, 315 nm); mobile phase, acetonitrile/tetrahydrofuran/distilled water (50,14,20, *v*/v/v); flow-rate, 1.6 mL/min.

**Results:**

The calibration curves obtained for colistin were linear in the concentration range of 0.10–8.0 μg/mL. The regression equation was *y* = 0.6496*×* − 0.0141 (*r*^2^ = 0.9999). The limit of detection was ~ 0.025 μg/mL, and the assay intra- and inter-day precisions were 0.87–3.74% and 1.97–6.17%, respectively. The analytical peaks of colistin A, colistin B, and the internal standard were resolved with adequate peak symmetries, and their retention times were approximately 8.2, 6.8, and 5.4 min, respectively. Furthermore, the assay was successfully applied to quantify the plasma colistin levels of a haemodialysis patient.

**Conclusion:**

The assay is a simple, rapid, accurate, selective, clinically applicable HPLC-based method for the quantification of colistin in human plasma.

## Background

Colistin, a well-known antibiotic, is a cationic polypeptide antimicrobial agent used for the treatment of Gram-negative pathogenic infections [1]. In recent years, these infections have become increasingly difficult to treat with standard agents because of the evolution of a wide variety of resistance mechanisms; hence, colistin has re-emerged as a treatment of choice for Gram-negative pathogens, including multidrug-resistant *Pseudomonas aeruginosa* (MDRP), a virulent hospital-acquired infection [2–6]. As the antibacterial effects of colistin are known to be concentration-dependent, several researchers have investigated the pharmacokinetic (PK) and/or pharmacodynamic profiles of colistin [7–9]. Colistin also exhibits a number of common adverse effects, including nephrotoxicity and neurotoxicity, which are also concentration-dependent. This has led to justifiable concerns that the current recommended dosage excessively increases the risk of such adverse effects in patients [10–12]. The establishment of a simple and rapid clinically applicable measurement system for assessing colistin concentrations is therefore of particular importance in hospitals. Such a system could then be employed to determine the appropriate dosage regimen for colistin therapy to ensure its efficacy and safety.

To date, several techniques have been developed for the measurement of colistin concentrations, including methods based on microbiological assays [13, 14], high-performance liquid chromatography (HPLC) combined with ultraviolet [15, 16] or fluorescence detection [17–19], capillary electrophoresis combined with laser-induced fluorescence detection [20], and liquid chromatography-mass spectrometry (LC-MS) [21, 22]. However, the majority of these methods have drawbacks. For example, microbiological assays lack selectivity and are overly time consuming. In addition, although LC-MS is a particularly accurate technique, it is expensive and unavailable in many hospitals. On the other hand, HPLC-based methods could be easily adopted in clinical practice. Although the HPLC method based on the use of fluorescence reagents is widely known, it does not offer satisfactory sensitivity, repeatability, and/or reliability, and is also rather time consuming in practice [19].

Therefore, we aimed to establish a rapid, simple, and clinically applicable HPLC-based measurement system using a short analytical column to determine colistin concentrations in hospitals. Furthermore, we examined the application of this system to measure the colistin concentration in a plasma sample obtained from a haemodialysis patient.

## Methods

### Instrumentation

The HPLC system consisted of a Shimadzu LC-10 AD pump (Kyoto, Japan) equipped with a Shimadzu RF-5300 fluorescence detector and a Shimadzu CTO-6A column oven. The analytical column was a reverse-phase Hydrosphere C18 column (internal diameter [i.d.], 4.6 × 50 mm, 5 μm), which was purchased from YMC Co., Ltd. (Kyoto, Japan).

### Chemicals and reagents

Analytical grade colistin sulfate, netilmicin sulfate, 9-fluorenylmethyl chloroformate (FMOC-Cl), trichloroacetic acid, sodium hydroxide, acetone, sodium hydrogen carbonate, and boric acid, and HPLC grade methanol, acetonitrile, tetrahydrofuran, and distilled water were purchased from Wako Pure Chemical Industries, Ltd. (Osaka, Japan). The serum employed for quality control (QC) was purchased from Alfresa Pharma Corporation (Osaka, Japan).

Stock solutions of colistin sulfate (100 μg/mL) and netilmicin sulfate (5 μg/mL) internal standard were prepared by dissolving 1.0 and 0.05 mg of the respective substances in 10 mL of distilled water. A 100 mM FMOC-Cl stock solution was prepared by dissolving 258.7 mg of FMOC-Cl in 10 mL of acetonitrile. The carbonate buffer (1 wt%, pH 10) was prepared by dissolving the sodium hydrogen carbonate (1 g) in distilled water (100 mL) and the pH of the solution was adjusted to 10 using sodium hydroxide. All solutions were stable for at least 2months when stored in a refrigerator at 4 °C.

### Chromatographic conditions

HPLC analysis was performed at 25 ± 1 °C under isocratic conditions. All measurements were carried out at excitation and emission wavelengths of 260 nm and 315 nm, respectively, and the column temperature was maintained at 40 °C. The mobile phase consisted of a mixture of acetonitrile/tetrahydrofuran/distilled water (50:14:20, *v*/v/v) and was delivered at a flow rate of 1.6 mL/min. The sample injection volume was 50 μL.

### Sample preparation

Initially, the colistin and the internal standard (netilmicin sulfate, 20 μL) stock solutions were added to a portion of serum (200 μL). Following the addition of methanol (25 μL) and 10% trichloroacetic acid (25 μL) to the sample, it was vortexed for 10 s prior to centrifugation at 13,000 rpm for 5 min. The supernatant was then placed in an additional centrifuge tube and mixed with a 1 M sodium hydroxide solution (10 μL). The sample solution was loaded onto the solid-phase extraction (SPE) cartridge, which had been previously conditioned according to the following procedure.

SPE C18 cartridges (55 μm, 100 mg/mL; Phenomenex, Torrance, USA) were conditioned using acetone (1 mL) and methanol (1 mL) and equilibrated with 1% carbonate buffer (1 mL). The sample was then applied to the wet cartridge. After passing the sample through the cartridge, it was rinsed with methanol (1 mL) and carbonate buffer (1 mL), followed by a 100 mM FMOC-Cl solution (60 μL) and 90% methanol (3 mL). The derivatives were then eluted into a glass culture tube using acetone (500 μL). The eluted solution was mixed with a 0.6 M boric acid solution (100 μL) prior to vortex-mixing for 10 s, and then the obtained sample was injected into the HPLC system. All procedures were conducted at 25 ± 1 °C.

### Establishment of a calibration curve

The colistin stock solution was added to the serum to obtain solutions of the following concentrations: 0.10, 0.25, 0.5, 1.0, 2.0, 4.0, and 8.0 μg/mL. The internal standard (20 μL) was then added to each sample and five measurements were taken at each concentration. The ratio of the colistin A and colistin B peaks to that of the internal standard was determined at each colistin concentration and used to produce a calibration curve. The least-squares method was used to calculate the calibration equation and the correlation coefficient, and to verify the regression.

The lower limit of quantitation (LLOQ) was defined as the lowest concentration of colistin that could be quantitatively determined with acceptable precision and accuracy. Acceptance limits were defined as an accuracy of 80–120% and a precision of < 20%. The limit of detection (LOD) was defined as the lowest concentration of colistin that could be distinguished from the blank with a signal-to-noise ratio (SNR) ≥3.

### Precision and accuracy

The precision and accuracy of the assay were evaluated by the assessment of the QC samples spiked with 0.1 (LLOQ), 0.50 (low QC), 2.0 (middle QC), 4.0 (high QC) μg/mL colistin in five replicates over three different validation days. Precision and accuracy were assessed by comparing the measured concentrations in the QC samples (five separately prepared sets measured on one day (intra-day), three different days (inter-day)) with the respective colistin concentrations, which were expressed as the respective coefficients of validation of the mean values (precision) and as the relative error (accuracy). The QC samples were spiked independently from the calibration standards using separately prepared stock solutions.

### Derivative stability

The derivative stabilities of the samples containing 0.10 and 8.0 μg/mL of the standard colistin solution and the internal standard were evaluated at − 23 (frozen), 4 (refrigerated), and 25 ± 1 °C (room temperature) over 7 d. Freeze-thaw stability was assessed after three complete freeze-thaw cycles (− 23 °C to room temperature) on consecutive days. The stability of the target compounds was presented as the recovery (%) relative to the freshly prepared samples. The solutions at room temperature were exposed to normal fluorescent light, while the frozen and refrigerated samples were stored in a dark refrigerator and were exposed to light only during sampling. The storage temperatures were closely monitored throughout the study.

### Robustness

To evaluate the robustness of the assay, the following variables were examined: pH of the carbonate buffer solution, concentration of the FMOC-Cl solution, and the reaction time in the presence of FMOC-Cl for the colistin derivatisation process.

### Application of the HPLC method to the plasma colistin sample of a haemodialysis patient

A 72-year-old female patient (weight 52 kg, height 153.2 cm) with antineutrophilic cytoplasmic antibody-positive vasculitis undergoing renal replacement (intermittent haemodialysis, HD) was admitted to the hospital for rituximab and steroid pulse therapy. The HD patient’s disease had been complicated by *Pneumocystis jiroveci* pneumonia, herpes zoster, and MDRP infections. Baseline conditions included: temperature, 39.1 °C; heart rate, 108 beats/min; respiratory rate, 22 beats/min; blood pressure, 160/80 mmHg; leukocytes, 11,300 /mm^3^; and C-reactive protein, 6.8 mg/dL. Blood cultures from two peripheral vein sites were positive for MDRP with metallo-beta-lactamase, and the minimum inhibitory concentrations (μg/mL) were as follows: colistin ≤1; piperacillin, 8; sulbactam-ampicillin, > 32; tazobactam-piperacillin, 32; ceftazidime, > 32; cefepime, > 32; imipenem-cilastatin, > 8; meropenem, > 8; gentamicin, > 16; amikacin, > 16; minocycline, > 8; ciprofloxacin, > 4; aztreonam, 16; and sulfamethoxazole-trimethoprim, > 80. With the preliminary information of positive blood cultures, empiric treatment with cefepime (1 g every 12 h) was performed over 5 d. As the final result was isolation of the abovementioned MDRP at day 6, antibiotic treatment was switched to colistin methanesulfonate (CMS, 75 mg every 24 h after a loading dose of 250 mg) in combination with meropenem (1 g every 24 h). In addition, the following treatment parameters were employed: dialysate flow rate, 500 mL/min; blood flow rate, 100 mL/min; membrane surface area, 1.5 m^2^; membrane type, APS-15MD New; HD frequency, twice per week; and session duration, 3 h. Samples were collected immediately prior to a dose or immediately prior to the next dose if CMS was not being administered every 24 h. The actual times of CMS administration and blood sampling were recorded. Samples were collected in potassium (K)_2_-ethylenediaminetetraacetic acid (EDTA)-containing tubes and centrifuged for 15 min within 2 h of collection. The resulting plasma sample was measured on the same day as blood collection. The HD clearance (CL_HD_) for colistin was calculated every hour during the start and end of the HD session as:

CL_HD_ = (Cp_pre_ − Cp_post_) / Cp_pre_ × Q_B_ × (1 − Ht).

where Q_B_ corresponds to the blood flow rate, Ht refers to haematocrit, and Cp_pre_ and Cp_post_ indicate the colistin concentrations at the start and end of the HD session, respectively.

The study protocol was approved by the research ethics committee of Toho University Omori Medical Center (Approval Number M17280).

## Results

### HPLC chromatograms

**Fig. 1:**
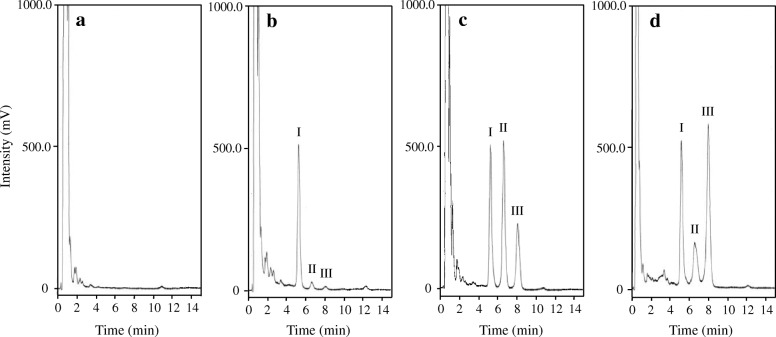
Typical chromatograms obtained via fluorescence-based (excitation at 260 nm, emission at 315 nm) detection of colistin. **a** Blank serum, **b** serum sample containing 0.10 μg/mL colistin, **c** serum sample containing 4.0 μg/mL colistin, and **d** plasma sample obtained from a haemodialysis patient. Peak I = netilmicin, peak II = colistin B, and peak III = colistin A

Representative chromatograms of the blank and spiked serum samples containing 0.10 and 4.0 μg/mL of the standard colistin solution in addition to the internal standard are shown in Fig. 1. As indicated, the peaks corresponding to colistin A, colistin B, and the internal standard were resolved with adequate peak symmetries, and the retention times of colistin A and colistin B were approximately 8.2 and 6.8 min, respectively, whereas that of the internal standard was approximately 5.4 min. No interference peaks were detected, and the target peaks were selectively isolated from the other serum components.

### Calibration curve

The calibration curve produced based on the ratio of the colistin A and colistin B peaks to that of the internal standard in serum samples (200 μL) containing 0.10–8.0 μg/mL colistin was linear (*y* = 0.6496*×* − 0.0141) in the examined concentration range and the correlation coefficient, *r*^2^, was 0.9999. Similarly, the individual calibration curves for colistin A and colistin B were also linear (colistin A; *y* = 0.1981*×* + 0.0040, colistin B; *y* = 0.4515*×* + 0.0101) in this concentration range, and both correlation coefficients were > 0.9999. The validation of these results is summarised in Table 1.

**Table 1:** Summary of the method validation results

Parameter	Result
Concentration range (μg/mL)	0.10–8.0
Regression equation^a^	*y* = 0.6496*×* − 0.0141
SE of slope^b^	0.0230
SE of intercept^b^	0.0019
Determination coefficient (*r*^2^)	0.9999
Intra-day precision (%)^c^	2.69
Inter-day precision (%)^c^	4.01
Intra-day accuracy (%)^c^	103.9
Inter-day accuracy (%)^c^	105.2
Lower limit of quantification (μg/mL)	0.10
Limit of detection (μg/mL)	0.025
Stability	acceptable
Robustness	acceptable

^a^*y* = a*x* + b, where *y* is the ratio of the summed peak areas of colistin A and B to that of the internal standard, and *x* is colistin concentration (μg/mL)

^b^SE, standard error (*n* = 3)

^c^Mean of four concentrations (0.10, 0.50, 2.0, and 4.0 μg/mL)

### Precision and accuracy

Table 2 shows our findings regarding the reproducibility of the repeated measurements obtained at four different colistin concentrations (0.10, 0.50, 2.0, and 4.0 μg/mL). The intra-day precision ranged from 0.87 to 3.74%, which was indicative of reproducibility of < 4%. The inter-day precision ranged from 1.97 to 6.17%, which was 1.5 to 2 times higher than the intra-day precision but still indicative of adequate reproducibility. The accuracy ranged from 99.0 to 115.0%, which was adequate within ±15% of the normal value. As the accuracy was within the acceptable range, the LLOQ and LOD were determined, giving values of 0.10 and 0.025 μg/mL, respectively, with an SNR of 4.

**Table 2:** Intra- and inter-day precisions of the assay for determining colistin concentration in the control serum

Colistin concentration (μg/mL)	Intra-day		Inter-day	
	Precision (%)	Accuracy (%)	Precision (%)	Accuracy (%)
High QC (4.00)	0.87	102.0	1.97	102.3
Middle QC (2.00)	2.64	99.0	4.19	101.9
Low QC (0.50)	3.74	100.7	3.71	101.4
LLOQ (0.10)	3.50	113.8	6.17	115.0

*QC*, quality control; *LLOQ*, lower limit of quantitation

### Derivative stability

The derivatives of colistin A, colistin B, and netilmicin (the internal standard) were relatively stable in the eluted solutions for the frozen, refrigerated, and room temperature samples stored over 7 d in closed glass test tubes (Table 3). Compared with the peak areas of the freshly prepared samples, the recoveries of the derivatives produced using 0.10 and 8.0 μg/mL solutions of colistin ranged from 100 to 105, 98 to 104, and 99 to 103% for the frozen, refrigerated, and room temperature samples, respectively. For the freeze-thaw stability, the recoveries after three complete freeze-thaw cycles on consecutive days ranged from 97 to 102%. There were essentially no degradation peaks and no new peaks were observed in the samples over the 7 d storage time or after freeze-thaw cycles.

**Table 3:** Stabilities of colistin A, colistin B, and netilmicin

Sample	Concentration (μg/mL)	Frozen^a^ (%)	Refrigerated^a^ (%)	Room temperature^a^ (%)	Freeze-thaw^b^ (%)
Colistin A	8.00	102 ± 2	100 ± 3	99 ± 2	100 ± 2
	0.10	105 ± 3	100 ± 1	102 ± 3	97 ± 3
Colistin B	8.00	100 ± 3	98 ± 3	101 ± 2	99 ± 3
	0.10	105 ± 2	102 ± 2	102 ± 2	102 ± 3
Netilmicin	8.00	103 ± 3	101 ± 2	103 ± 2	102 ± 2
	0.10	102 ± 3	104 ± 2	102 ± 3	98 ± 3

^a^Samples were stored over 7 d

^b^Sample were frozen at −23 °C and thawed at room temperature for three cycles

Data are reported as the mean ± standard deviation

Frozen, − 23 °C; Refrigerated, 4 °C; Room temperature, 25 ± 1 °C

### Robustness

**Fig. 2:**
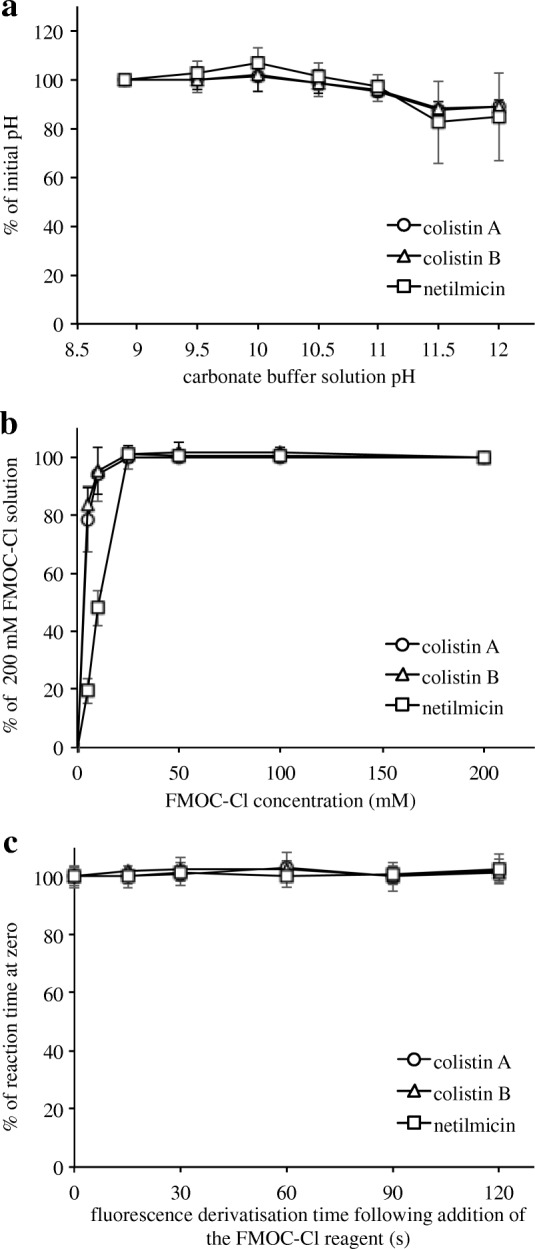
Optimisation data for the colistin derivatisation process. Variation in the (**a**) carbonate buffer solution pH, **b** FMOC-Cl concentration, and (**c**) fluorescence derivatisation time following addition of the FMOC-Cl reagent. Data are presented as the mean ± standard deviation

To demonstrate the robustness of the assay, a series of variables was employed during the derivatisation of colistin with FMOC-Cl. More specifically, the pH of the carbonate buffer solution was varied from a value of 8.9 prior to the addition of sodium hydroxide. As shown in Fig. 2a, no increase or decrease in the colistin A, colistin B, and netilmicin peak areas were observed upon increasing the solution pH to 11 (i.e., between pH 8.9 and 11), while an increase to pH 11.5 or higher led to a > 10% reduction in the peak area. In addition, six different FMOC-Cl concentrations were examined, ranging from 5.0 to 200.0 mM. Upon increasing the FMOC-Cl concentration to ~ 25 mM, the peak areas of colistin A, colistin B, and netilmicin increased significantly (Fig. 2b). However, at higher FMOC-Cl concentrations, no further increase in the peak area was observed. Furthermore, as shown in Fig. 2c, the peak areas of colistin A, colistin B, and netilmicin ranged from 95 to 105% of the peak areas at time = 0.

### Application to the plasma colistin sample of a haemodialysis patient

**Fig. 3:**
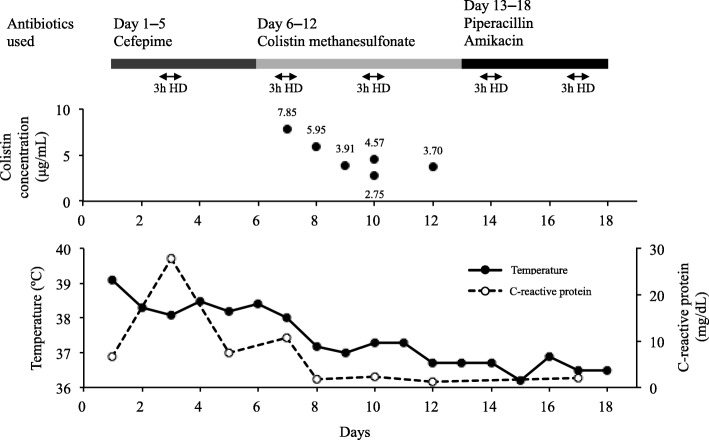
Clinical course and antimicrobial therapy for a haemodialysis patient suffering from a multidrug-resistant *Pseudomonas aeruginosa* infection. HD = intermittent haemodialysis

CMS was administered to the HD patient over 7 d (days 6–12), and six samples of blood were collected between days 7 and 12 of CMS therapy (Fig. 3). The initial sample was collected on day 7 approximately 20 h after the administration of CMS. All other samples were collected ~ 24 h after each dose. On the day of dialysis, the blood samples were collected at the start and end of the HD session.

As shown in Fig. 1d, this method was successful in its application to the measurement of the plasma colistin of the HD patient. In addition, although the patient was administrated with meropenem, trimethoprim-sulfamethoxazole, fluconazole, prednisolone, nifedipine, acetaminophen, alendronate, and esomeprazole in combination with colistin for the treatment of both the MDRP infection and comorbid diseases, no interference peaks were detected in the chromatogram and the target peaks were selectively isolated. The colistin concentration on day 7 was 7.85 μg/mL, and those on days 8, 9, and 12 were 5.95, 3.91, and 3.70 μg/mL, respectively. Furthermore, the colistin concentration at the start and end of the HD session and haematocrit value on day 10 were 4.57 and 2.75 μg/mL, and 22.9%, respectively; hence, the removal rate of colistin following HD (a 3 h session) was calculated to be 39.8% and its CL_HD_ was 30.7 mL/min.

Regarding the clinical course for the HD patient, the fever subsided, the C-reactive protein level decreased, and blood cultures were found to be negative after a few days of switching to CMS therapy (Fig. 3). However, CMS treatment was discontinued after 7 d because of the risk of adverse effects, and antibiotic treatment was substituted with piperacillin (3 g every 12 h) and amikacin (500 mg every 24 h). As the patient’s condition remained good after changing to this regimen, treatment of the MDRP infection could be considered successfully completed in a total of 18 d. The patient was finally cured without any apparent adverse effects during antibiotic therapy.

## Discussion

In this study, a rapid, simple, and novel HPLC-based method involving the use of a short analytical column was developed to quantify the concentration of colistin in serum samples. The HPLC run time was ~ 9 min, which, to the best of our knowledge, is the fastest HPLC-based method for the quantification of colistin in human plasma.

As colistin exhibits an extremely weak ultraviolet absorption and does not produce native fluorescence, the use of fluorescence reagents is necessary when determining colistin concentrations using HPLC-based methods. In previous colistin assays, *ortho*-phthalaldehyde (OPA) has been employed as the derivatising reagent; however, the reaction conditions for this transformation must be carefully controlled. An HPLC system equipped with a precolumn and an analytical column are typically required because of the instability of the obtained derivatives [17, 18]. Therefore, we attempted to develop a colistin assay based on FMOC-Cl as the derivatising reagent. Indeed, sufficiently stable derivatives were obtained over 7 d using milder reaction conditions. In addition, as reported by Decolin et al. that the optimal period between OPA addition and injection was 1–2 min [17], we believe that our method is superior to previously reported methods in the context of its higher stability.

Subsequently, to reduce the analytical time required, we utilized a short analytical column for the separation of colistin. We initially examined several conventional analytical columns, including Develosil ODS-UG-5 (i.d., 4.6 × 250 mm, 5 μm) similar Li’s group [19]; however, these columns produced broad colistin peaks, and the HPLC run times were considerable. Indeed, Li et al. reported that retention times of 26.1 and 21.8 min were recorded for colistin A and colistin B, respectively [19]. Therefore, for the purpose of this study, the reversed-phase Hydrosphere C18 column (i.d., 4.6 × 50 mm, 5 μm) was selected as it yielded shorter analysis times, in addition to a superior selectivity and sensitivity. Furthermore, we evaluated the mobile phase composition based on previous reports [19, 23] that it was necessary to add small quantities of tetrahydrofuran to the mobile phase. However, we found that this resulted in the poor separation of colistin from the internal standard, as the colistin retention time was too short (i.e., < 3 min). We therefore adopted an isocratic system of acetonitrile/tetrahydrofuran/distilled water (50:14:20, *v*/v/v), which produced sharp and well-separated colistin peaks. Furthermore, the HPLC run time of our assay was only 9 min, which was significantly shorter than that reported by Li et al. [19].

We also examined the development of a simple and practical sample preparation process. Thus, following deproteinisation, the centrifugation time was further reduced compared with previous colistin assays by employing high-speed centrifugal fractionation and an organic solvent [19, 23]. In addition, in the derivatisation of colistin using FMOC-Cl, manifold drying was omitted and the quantity of the reaction product eluate added to the samples was reduced. We found that the resulting method exhibited an adequate precision and accuracy following the rapid (10 min) sample preparation process compared with the longer preparation times (i.e., > 30 min) required by previous colistin assays [19, 23].

We successfully applied this assay to patient plasma samples to quantify the levels of colistin and demonstrated that the developed assay could be used for therapeutic drug monitoring in hospitals. Interestingly, no adverse effects such as neurotoxicity were observed during CMS therapy over 7 d through management of the colistin concentration between 3.70 and 4.57 μg/mL. In this context, Sorlí et al. have reported that the peak and minimal concentrations of colistin for patients suffering from nephrotoxicity are approximately 0.16–6.12 μg/mL (median 1.81 μg/mL) and 0.16–5.99 μg/mL (median 1.18 μg/mL), respectively [24], while Garonzik et al. reported that the average steady-state concentration of colistin for critically ill patients, including those on HD and continuous renal replacement, was 0.48–9.38 μg/mL (median, 2.36 μg/mL) [25]. These results indicate that there is a considerable inter-study variation in the colistin concentration following CMS therapy among patients with chronic renal dysfunction or undergoing HD. Furthermore, Sorlí et al. showed nephrotoxicity rates of 65–85% with trough concentrations > 2.2 μg/mL [24], while Garonzik et al. suggested an average target concentration of 2.5 μg/mL based on the population PK model [25]. These results were suggested as a compromise between efficacy and toxicity. Hence, we reaffirmed that the therapeutic drug monitoring of colistin is important to clarify the most appropriate dosage regimen in colistin therapy to ultimately ensure its efficacy and safety, especially for patients with renal dysfunction or undergoing HD.

We also found that colistin was removed efficiently by HD over 3 h (dialysis removal rate, ~ 40%; CL_HD_, 30.7 mL/min). Previously, Marchand et al. and Garonzik et al. also reported that the time-averaged dialysis clearances of colistin during HD were ~ 134–140 mL/min and 3.40 L/h, respectively [25, 26]. We consider that the differences in the CL_HD_ of colistin between our study and previous studies were because of the HD parameters employed, including the dialysate flow rate, blood flow rate, membrane type, and session duration; however, the influence of HD on colistin treatment remains unclear owing to the limited number of reports available.

We should also point out that our study had some limitations. First, our HPLC-based method exhibited lower colistin sensitivity than LC-MS methods. However, the linearity ranges from 0.1 to 8.0 μg/mL of our assay adequately covered the therapeutic ranges of colistin when used as an antibiotic in clinical practice. Naturally, HPLC is relatively inexpensive and available in many hospitals. Therefore, this study focused on the development of a rapid and simple HPLC-based system for the clinical determination of colistin concentrations in a hospital setting. Second, we evaluated the application of this assay to measure the colistin concentration in only a single patient. Third, CMS was only administered over 7 d, which could be considered a relatively short treatment time. We therefore consider that it is necessary to gather additional clinical data on our rapid and simple HPLC-based method for the quantification of colistin, and this will be addressed in the near future in our research group.

## Conclusion

We successfully developed a rapid and simple HPLC-based system for the clinical determination of colistin serum concentrations. Furthermore, our assay was successfully applied to the analysis of a plasma sample from an HD patient. Thus, this assay is useful for determining the optimal and safe dose of colistin required for the treatment of patients with chronic renal dysfunction or undergoing HD, for whom the administration of colistin in hospitals has been challenging.

## Abbreviations

CL_HD_: Ntermittent haemodialysis clearance
CMS: Colistin methanesulfonate
Cp_post_: Colistin concentrations at end of intermittent haemodialysis session
Cp_pre_: Colistin concentrations at the start
EDTA: Ethylenediaminetetraacetic acid
FMOC-Cl: 9-fluorenylmethyl chloroformate
HD: Intermittent haemodialysis
HPLC: High-performance liquid chromatography
LC-MS: Liquid chromatography-mass spectrometry
LLOQ: Lower limit of quantitation
LOD: Limit of detection
MDRP: Multidrug-resistant *Pseudomonas aeruginosa*
OPA: *Ortho*-phthalaldehyde
PK: Pharmacokinetic
Q_B_: Blood flow rate
QC: Quality control
SNR: Signal-to-noise-ratio
SPE: Solid-phase extraction
